# Screening for antenatal depression and its determinants among pregnant women in Qatar: revisiting the biopsychosocial model

**DOI:** 10.1186/s12884-021-03793-7

**Published:** 2021-04-26

**Authors:** Sarah Naja, Noora Al Kubaisi, Rajvir Singh, Hiba Abdalla, Iheb Bougmiza

**Affiliations:** 1 Community Medicine Residency Program, Department of Medical Education, Hamad Medical Corporation, P.O. Box 3050, Doha, Qatar; 2 Primary Health Care Corporation, Doha, Qatar

**Keywords:** Antenatal depression, Screening, EPDS

## Abstract

**Background:**

Screening for antenatal depression and its determinants is highly recommended. However, there is no consensus on a standard conceptual framework to approach pregnant women in a primary health care setting. The prevalence of antenatal depression and significant determinants are unknown in Qatar, a gap that we propose to fill.

**Methods:**

An analytical cross-sectional study with a probability sampling technique was employed. Enrolling eight-hundred participants from primary health care centers. The screening was performed through a valid and reliable screening instrument ‘Edinburgh Postpartum Depression Scale.’ In addition to the proposed Comprehensive Biopsychosocial Model, participants were asked about their predisposing profile, biological risk, and other suggested modifiable variables.

**Results:**

Twenty-one percent reported minor depressive episodes (*n* = 167, 20.9%) at a 95% confidence interval [18.2–23.8]. Previous use of mental health medications, fear of giving birth, concern about appearance, low perceived social support, and low resilience level showed to be associated with antenatal depression. The logistic regression analyses revealed that the Comprehensive Biopsychosocial model forecast 89% of antenatal depression predictors provided Area Under the Receiver Operating Characteristic Curve of 0.89 at a 95% confidence interval [0.85 to 0.92].

**Conclusions:**

Antenatal depression is common among pregnant women in Qatar, and preventive interventions must target the determinants revealed. From a clinical perspective, the use of the proposed model may aid in the standardization of the screening process.

## Background

Depression among pregnant women is considered an early indicator of deterioration in the expecting mother’s mental health and the future generation’s developmental cascade [1, 2]. Accumulative evidence suggests an association between depression among pregnant women and morbid outcomes such as premature birth, low-birth weight, emergency C-Section, and a delay in breastfeeding initiation [3–5]. The economic burden of antenatal depression is five times higher than improving the service [6]. As a result, the United State Preventive Task Force (USPSTF) extended its recommendation to include screening for the associated factors of antenatal depression (history of depression, significant adverse life events, low income, and intimate partner violence), given that screening for antenatal depression alone proved to be insufficient. However, to date, no pragmatic approach was implemented to detect significant antenatal depression determinants [7].

Diverse conceptual models explained perinatal depression while few discussed antenatal depression as a unit acknowledging pregnancy stressors are different from those associated with baby arrival. For instance, the stress-vulnerability and stress-coping model was adopted among Korean pregnant women. However, this framework was criticized as it focused mainly on marital dissatisfaction and lacked acknowledging other social and behavioural determinants of women’s health [8].

The aetiology of depression among pregnant women is debatable and not fully understood. It has been hypothesized that pregnancy induces an inflammatory reaction contributing to perinatal depression [9]. However, this theory was previously announced in the biomedical model. It was criticised for pathologizing women’s reproductive biology and neglecting the impact of other external factors such as culture or society [10]. Several researchers found the Biopsychosocial model, developed by George L. Engel in 1977, to best explain the aetiology of perinatal depression [11]. Briefly, the Biopsychosocial paradigm suggests that biological factors do not directly trigger depression in pregnancy but rather indirectly initiate it through other intermediate variables such as psychosocial stressors and generalized anxiety [12].

Later in 2008, researchers adapted the Biopsychosocial model. Their findings similarly suggested that antenatal stressors, lack of personal resources, and low sociodemographic factors directly contributed to antenatal depression [13]. Moreover, a recent study in 2018 expanded the Biopsychosocial model to emphasize the significant role of unhealthy lifestyle as a covariate influencing antenatal depression [14]. Altogether, these findings highlight the Biopsychosocial model as the most widely accepted model to explain the aetiology of depression during pregnancy.

**Fig. 1:**
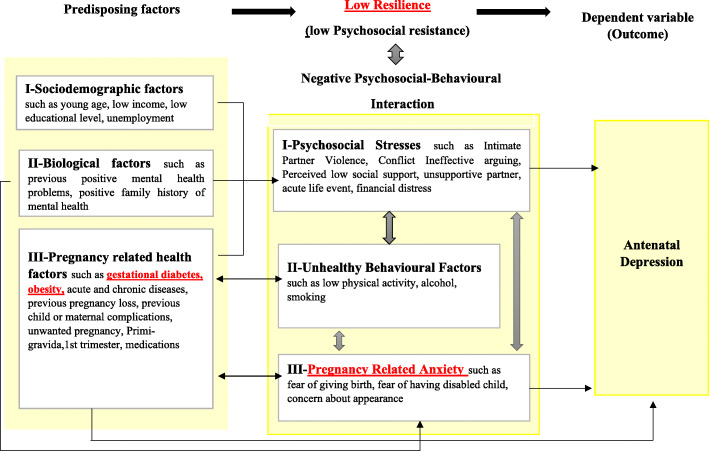
Comprehensive Biopsychosocial Conceptual Framework of Antenatal Depression and its Associated Factors at Primary Health Care Centers in Qatar

Ensuring healthy pregnancies is one of the crucial targets listed in Qatar’s National Strategy by 2022 [15]. To date, screening for antenatal depression is yet to be implemented. Reaching a consensus on a specific, pragmatic approach to detect antenatal depression and its determinants is necessary to standardize screening procedures and identify risk-groups. Based on the principles of general systems theory, we utilized the lens of the Biopsychosocial model [11]. This model builds on previous models and acknowledges the role of sociodemographic factors, psychosocial elements, and individual biology [12–15]. In addition, we highlighted intermediate modifiable factors, namely resilience and negative psychosocial-behavioural interaction, for their crucial role in adjusting and regulating antenatal depression, as supported by recent evidence [16, 17]. Moreover, we expanded on the potential contributing factors by adding perceived social support and pregnancy-specific anxiety, the latter being a distinctive syndrome thought to contribute to depression in pregnancy [18–20]. The proposed ‘Comprehensive Biopsychosocial Model’ in Fig. 1.

### Purpose

We aimed to determine the prevalence of antenatal depression among pregnant women in Qatar and investigate its determinants based on the proposed Comprehensive Biopsychosocial Conceptual Framework.

## Methods

### Study design and setting

We described an analytical cross-sectional study among pregnant women visiting antenatal clinics at the Primary Health Care Corporation (PHCC) in Qatar. Data was collected for 6 months from September 2018 to February 2019 from nine health centers’ morning (7:00 am to 2:00 pm) and evening shifts (4:00 pm to 10:00 pm) so that employed pregnant women would not be missed. The PHCC provided promotive and preventive health care services together with feasible and accessible care to Qatar’s national and expatriate communities, functioning through twenty-three primary health care centers at the time of the study. Antenatal support classes were not available in the PHCC at the time of the study. Importantly, these centers are considered the primary line of contact between the community and health care services; high- and low-income individuals visit them with an antenatal participation rate that is as high as 60% of the total live births. The rest of the pregnant women follow-up their antenatal care in the private sector, while others travel to their home country for medical care [15].

### Study population and sampling technique

A one-stage cluster sampling technique with proportional allocation was employed in this study [21]. First, the list of all the Primary Health Care Centers that provide antenatal clinic was obtained from the management of “Operations Department” in PHCC, which includes the total number and percentage of pregnant women attending in all and each health center in Qatar (twenty-three centers during the study period, total *n* = 6637 pregnant women aged 15–48 registered in all PHCCs, at the start of the study period). Next, the random sampling technique was conducted through Automated Random Number Generator to select nine health centers out of twenty-three Primary Health Care Centers. Each of the selected healthcare centers was designated as a cluster. Later, a proportionate allocation of participants from each of the nine selected primary healthcare centers was calculated based on the percentage of pregnant women attending antenatal clinic at Primary Health Care Centers.

The antenatal clinics at the selected centers were visited daily. The principal investigator or data collector explained the study to pregnant women. Participants who expressed interest in taking part in the study were screened for their eligibility. Non-random selection of pregnant women aged 15 to 49 years who met the inclusion criteria, which was not restricted to a specific trimester but involved communicating in Arabic and English and willingness to sign the consent and accent. Data collection continued until each health center fulfilled the calculated sample size.

### Sample size and enrolment of participants

The calculation was done to obtain a sufficiently precise estimate of the study sample and the study power. There are no studies that have previously explored the prevalence of depression among pregnant women in Qatar to the best of our knowledge. Therefore, we have used the prevalence of antenatal depression in Saudi Arabia (57.5%) since it is a regional Arab country that shares similar sociodemographic factors with Qatar [22].

The level of confidence used was 95%, and the acceptable error rate was 5% [23].

$$ \mathrm{n}=\left\{\left[{{\mathrm{Z}}^2}_{1-}{\propto}_{/2}\mathrm{x}\ \mathrm{p}\ \mathrm{x}\ \left(1-\mathrm{p}\right)\right]/{{\mathrm{d}}^2}_{\mathrm{x}}\ \mathrm{Design}\ \mathrm{Effect}\right\} $$

The estimated minimum sample size based on this calculation was (*n* = 726). Inflation of the sample (10%) was added to compensate for any non-response, so the total sample size targeted was (*n* = 800).

### Data collection

Eligible participants were first informed about the study through a research information sheet. Upon agreeing to participate and sign a written informed consent and accent form, participants were guided through an interviewer’s administered questionnaire about their sociodemographic characteristics, pregnancy-related characteristics, health behaviour, life stressors, intimate partner violence, and medical history, including mental health history. Next, anthropometric measures were taken for pregnant women in their first trimester. Data collectors reviewed all participants’ pregnancy-related notebook, including medical history, medications, lab results, and body mass index specifically for participants in their second and third trimesters. Following the interviewer-administrated questionnaire, participants were asked to fill the self-administrated tools on their own. Later, the EPDS scores were computed for pregnant women. Participants who showed any signs of suicidal thoughts (item 10) or scored 13 or above were referred to mental health specialized services.

### Variables and measures

#### Antenatal depression (dependent variable)

Antenatal depression, also known as prenatal depression, a mood disorder occurring during pregnancy. Several useful screening tools are available. However, researchers are against the inclusion of constitutional symptoms (e.g., changes in sleeping patterns and food habits) in screening antenatal depression because they are uninformative and non-specific (common in normal pregnancy). Thus, their inclusion may overestimate the disease’s actual burden [24, 25].

Edinburgh Postpartum Depression Scale (EPDS) is a self-report tool that consists of ten questions, and it does not include somatic symptoms. It can be completed in less than 5 min. Responses are scored 0, 1, 2, or 3 according to the symptom’s severity. The participants were asked to check one of four possible responses that mostly reflects how they have been feeling in the past 7 days. Previous studies suggest that this tool demonstrated good validity in English. Cut-off 13 revealed a sensitivity of 100% and a specificity of 87% (False Positive rate = 0.13) when tested among pregnant women [26]. A cut-off of 13 appeared to be valid in the Arabic language. It exhibited a sensitivity of 87% and a specificity of 90% with a Positive Predictive Value of 77%. Furthermore, it demonstrated a high Area under the Curve of 0.951 and good reliability (Cronbach’s alpha =0.865) in Qatar [27].

#### Determinants (independent variables)

Determinants were categorized based on the proposed Comprehensive biopsychosocial factors:

Predisposing factors included sociodemographic factors, biological factors, and pregnancy-related health conditions assessed through interviewer-administrated questionnaire. It included sociodemographic characteristics (nationality, age, family size number, household income, occupational status, and educational level), pregnancy-related characteristics (gravity, parity, number of children, gestational age, the desire of getting pregnant and infertility history). As well as, acute and chronic medical problems in pregnancy, behavioural factors (smoking, alcohol, and physical activity), stress-related factors (life stressful events, financial distress, partner social support, and lack of postpartum social support), mental health history (previous mental health disease, use of mental health-related medications, and family history) and anthropometric measures.

Intermediate factors included all stressors related to pregnancy and social factors:

Pregnancy-related anxiety is a distinctive syndrome that occurs in pregnancy [18]. Anxiety was assessed through the ten items Pregnancy-Related Anxiety Revised Version Two (PRAQ-R2). It is a self–report questionnaire consisting of ten items and three domains: fear of giving birth, fear of bearing a physically or mentally disabled child, and concerns about one’s appearance. It has been used in pregnancy for both multiparous and nulliparous women and was reported to have a good internal consistency (alpha > 0.8). Each item asked about feelings at present and assessed the frequency of the extent to which the factor was relevant based on 5- point Likert-type scale [28].

Perception of social support is a crucial psychological construct and relevant to individual cognition, sense of acceptance, and effective adjustment to stress. It was assessed through the perceived Social Support Questionnaire. The Short Version (F-SozU K-6) is a self-administrated tool initially developed in the German language. The questionnaire consists of six items on a 5-point Likert-type scale*.* F-SozU K-6 demonstrated high internal consistency (Cronbach’s alpha 0.94) and presented a valid and economically viable instrument to evaluate perceived practical, emotional, and social support systems [29].

Marital conflict is a state of stress or tension between the couple or married partners as they try to carry out their marital roles’ and sometimes known as high levels of disagreement [30]. It was assessed through the Ineffective Arguing Inventory, an English self-administrated tool used to measure marital conflict as an independent variable. It is an eight-question tool assessed on a 5-point Likert-type scale. Adding together the scores for each of the eight items determined the total score ranging between 0 (low/little arguing) to 32 (high/many arguments). The higher the score on this inventory, the more severe a marital conflict is. This tool was reported to have high internal consistency (alpha ranges from 0.86 to 0.89) [31].

Intimate partner violence is described as ‘physical violence, sexual violence, stalking and psychological aggression by a current or former intimate partner’ [32]. Hurt, Insult, Threaten, and Scream (HITS) measure intimate partner violence. It is a four-item tool assessed on a 5- point Likert-type scale. It was initially developed in the English language and the total score range between 4 and 20*.* Based on a systematic review, we categorized the scores into bivariate categories through the cut-off score > 10 that suggests a partner’s abuse. This cut-off point was revealed to have a pooled sensitivity of 86% and a pooled specificity of 99% (Positive Predictive Value 86% and Negative Predictive Value 99%) [33]. Additionally, participants screened positive were advised to seek social support services based on the Primary Health Care guideline for social help.

Resilience Scale (RS-11) was utilized to measure resilience as an independent variable defined as a dynamic modifiable process. It is the stable ability of a person to modulate and control one’s affective state and adequately adjust to burdens. It involves attributes of reintegration, self-determination, flexibility, sense of humour, and self-efficacy. RS-11 is the German short version of the Resilience Scale a 7-point Likert-type scale. It is a self-administrated tool that captures psychosocial stress-resistance. The scores can range from 0 (low resilience) to 32 (high resilience). It was reported to have a high internal consistency of Cronbach’s alpha 0.91 [34].

### Analysis

The database was constructed using the *Statistical Package for Social Sciences (SPSSTM)* software Version 23. The data were entered into a password-protected computer. Also, data cleaning was performed to check for any inconsistencies, errors, or redundancies. After which, the analysis was conducted and included three steps. Firstly, the descriptive statistics were tabulated in frequencies and percentages for categorical variables or mean ± standard deviation (Sd) for continuous variables. Then, the Kolmogorov test and Shapiro Wilk test were employed to assess the distribution of the dependent variable (EPDS scores-continuous variable) and were followed by bootstrapping. The domain scores for the following four tools (PRAQ-R2, F-SozU K-6, ineffective arguing inventory, and RS-11) were calculated through the sum of all the feasible items in each domain. The total was then divided by the number of items and multiplied by 100. A cut-off score of > 75 percentile serves as an indicator of a high level for each of the tools mentioned above. Secondly, the bivariate analyses were carried out using Chi-square tests. Then, Spearman’s rank correlation coefficient was employed to check for multicollinearity between the variables. Lastly, the significant variables for depression identified from the bivariate analyses were included in the multivariable logistic regression model. The association’s effect size was computed in adjusted odds ratios with a 95% CI and a *P*-value of 0.05 (two-tailed). The predictive probabilities from the final regression model were used for the Receiver Operating Curve (ROC). Sensitivity, Specificity, and c-statistics with 95% CI were presented in the ROC curve. We computed the reliability of the utilized tools through Cronbach’s alpha.

## Results

### Sample realisation

**Fig. 2:**
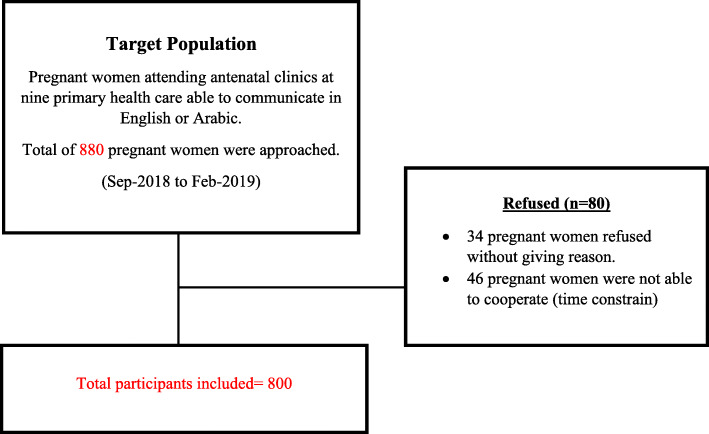
Flow chart of the study recruitment of the participants

During the period of data collection, a total of 880 pregnant women were approached to participate in the study. Eighty out of Eight hundred eighty pregnant women refused to participate due to lack of time. The non-response rate showed to be 9%, as seen in Fig. 2.

### Demographics and clinical profile

The average age of participants was 28.8 years (sd 5), 16–43. The predominating age group was between 20 and 34 years representing (*n* = 708, 88.5%). As for the nationalities, the majority of participants were non-Qatari (*n* = 655, 81.9%). More than half of the pregnant women had a higher education level (*n* = 548, 68.5%), and the majority (*n* = 529, 66.1%) were housewives.

As for the participant’s clinical profile, about two-thirds (*n* = 539, 67.4%) of the sample were multigravida. Approximately half of the participants were in their second trimester (*n* = 391, 48.9%). Additionally, one-fourth of the sample were obese (*n* = 262, 32.7%), with a mean weight of 65.5 kg (sd 13.5), and one-third of the participants were diagnosed with gestational diabetes in their current pregnancy (*n* = 261, 32.5%).

### Antenatal depression

The calculated mean EPDS scores were 7.26 (sd 5.7), 6–14, and the Std. Error of Mean is .2. The EPDS score distribution was moderately asymmetrical to the right, with a skewness coefficient of 0.64 and a kurtosis less than zero (− 0.32) indicating light tail (platykurtic distribution). Additionally, the bootstrap results were based on the central tendency theory. The normally distributed sample demonstrated zero bias and the true mean value is 6 [6.9–7.7] at 95% CI.

The proportion of pregnant women with antenatal depression (EPDS ≥13) was (*n* = 167, 20.9% (95% CI [6.8 to 7.6])). A total of (*n* = 108, 13.5%) participants scored zero, and the 90th percentile was 16. Specifically, a total of (*n* = 532, 66.5%) participants scored from zero to 9 while (*n* = 101, 12.7%) scored between 10 to 12, demonstrating low risk response in question 10**.** Feeling overwhelmed was the most reported depressive symptom among pregnant women, where (*n* = 63,7.9%) scored three by answering ‘Yes, most of the time I haven’t been able to cope at all’ in item 6, which is ‘Things have been getting on top of me.’ As for suicidal thoughts, only a few participants (*n* = 3,0.4%) reported its presence, whereby they score three by answering ‘quite often’ in item 10, which is ‘The thought of harming myself has occurred to me.’

### Determinants

Table 1 includes predisposing characteristics that could potentially be associated with antenatal depression. The results suggest that older age, multigravida, second trimester, overweight or obese, low household income, and a positive history of mental illness medications were all associated with antenatal depression.

**Table 1:** Antenatal depression and its association with the predisposing characteristics presented in the Comprehensive Biopsychosocial Model (*n* = 800)

Predisposing Characteristics	EPDS		Total n (%)	χ2	***p*** value
	≥ 13 Depressed n (%)	< 13 Not Depressed n (%)			
**I-Sociodemographic factors**					
Age^*^					
15–19	5 (3.0)	19 (3.0)	24 (3.0)		
20–34	127 (76.0)	534 (84.4)	661 (82.6)	7.4	0.021^*^
35–46	35 (21.0)	80 (12.6)	115 (14.4)		
**Nationality**					
Non-Qatari	139 (83.2)	516 (81.5)	655 (81.9)	0.26	**0.610**
Qatari	28 (16.8)	117 (18.5)	145 (18.1)		
**Educational level**					
Higher education	114 (68.7)	434 (68.5)	548 (68.5)		
Secondary education	37 (22.3)	149 (23.5)	186 (23.2)	2.0	**0.551**
Primary education	10 (6.0)	42 (6.6)	52 (6.5)		
Illiterate	5 (3.0)	9 (1.4)	14 (1.8)		
**Occupational Status**					
Housewife	113 (67.7)	416 (65.7)	529 (66.1)		
Employed	54 (32.3)	217 (34.3)	271 (33.9)	0.2	**0.611**
Family size^*^					
Small family size (< 5)	74 (44.3)	403 (63.7)	477 (59.6)	20.5	0.000^*^
Large family size (≥ 5)	93 (55.7)	230 (36.3)	323 (40.4)		
Household Monthly income^*^					
< 10.000 QR	72 (43.1)	285 (45.0)	357 (44.6)	6.5	0.039^*^
10.000–20.000 QR	69 (41.3)	204 (32.2)	273 (34.1)		
> 20.000 QR	26 (15.6)	144 (22.8)	170 (21.3)		
**II-Pregnancy-Related Characteristics**					
Gravity^*^					
Primigravida	42 (25.1)	219 (34.6)	261 (32.6)	5.3	0.021^*^
Multigravida	125 (74.9)	414 (65.4)	539 (67.4)		
Trimesters^*^					
1st trimester	53 (31.8)	144 (22.7)	197 (24.6)	7.8	0.021^*^
2nd trimester	67 (40.1)	324 (51.2)	391 (48.9)		
3rd trimester	47 (28.1)	165 (20.1)	212 (26.5)		
**Previous abortion**					
Yes (< 20 weeks gestation)	40 (24.0)	118 (18.6)	158 (19.8)	2.3	**0.122**
No	127 (76.0)	515 (81.4)	642 (80.2)		
**Previous C-Section**					
Yes	41 (33.9)	104 (26.7)	145 (28.4)	2.3	**0.121**
No	80 (66.1)	285 (73.3)	365 (71.6)		
Body Mass Index^*^					
Underweight	1 (0.6)	17 (2.7)	18 (2.25)	18.5	0.011^*^
Normal	47 (28.1)	233 (36.8)	280 (35.0)		
Overweight	42 (25.1)	198 (31.3)	240 (30.0)		
Obese	77 (46.2)	185 (29.2)	262 (32.75)		
**III-Biological psychiatric risk**					
**Previous use of medications related to mental illness** (more than 6 times in any year)					
Yes	9 (5.4)	1 (0.2)	10 (1.25)	29	0.000^*^
No	157 (94.6)	633 (99.8)	790 (98.75)		
**Positive family history of mental health illness**					
Yes	10 (6.0)	22 (3.5)	32 (4.0)	2.1	**0.441**
No	157 (94.0)	611 (96.5)	768 (96.0)		

*EPDS* Edinburgh Postpartum Depression Scale, *QR* Qatari Ryal

^*^p ≤ 0.05

χ^2^ = Chi-square

Most of the negative psychosocial stresses showed to be significant determinants for antenatal depression. It is noteworthy that being stressed about baby gender or being a smoker were not significant determinants, as shown in Table 2.

**Table 2:** Antenatal depression and its association with the intermediate variables presented in the Comprehensive Biopsychosocial Model (*n* = 800)

Intermediate Variables	EPDS		Total n (%)	χ2	***P*** value
	≥ 13 Depressed n (%)	< 13 Not Depressed n (%)			
**I-Psychosocial stressful factors**					
Stressful Life events^*^					
Yes	16 (9.6)	22 (3.5)	38 (4.75)	10	0.001^*^
No	151 (90.4)	611 (96.5)	762 (95.25)		
**Stressed about baby gender**					
Not at all	99 (59.2)	424 (67.0)	523 (65.4)		
Very little	14 (8.4)	62 (9.7)	76 (9.5)	6.5	**0.081**
Somewhat	20 (12.0)	46 (7.3)	66 (8.3)		
To a great extent	34 (20.4)	101 (16.0)	135 (16.8)		
Financial distress^*^					
High	14 (8.4)	18 (2.8)	32 (4.0)	10.5	0.011^*^
Low	153 (91.6)	615 (97.2)	768 (96.0)		
Perceived Social support^*^					
High	78 (46.7)	519 (82.0)	597 (74.6)	86	0.000^*^
Low	89 (53.3)	114 (18.0)	203 (25.4)		
Lack of husband’s emotional support^*^					
Yes	47 (28.1)	21 (3.3)	68 (8.5)	104	0.000^*^
No	120 (71.9)	612 (96.7)	732 (91.5)		
Lack of future postpartum support^*^					
Yes	38 (22.8)	69 (10.9)	107 (13.4)	16	0.000^*^
No	129 (77.2)	564 (89.1)	693 (86.6)		
Marital Conflict^*^					
High	33 (19.8)	24 (3.8)	57 (7.1)	50	0.000^*^
Low	134 (80.2)	609 (96.2)	743 (92.9)		
Intimate partner violence^*^					
Yes	12 (7.2)	1 (0.15)	13 (1.6)	40.8	0.000^*^
No	155 (92.8)	633 (99.85)	787 (98.4)		
**II-Unhealthy Behaviour**					
Fitness^*^					
Not fit	143 (85.6)	417 (65.8)	560 (70.0)	25	0.000^*^
Active	12 (7.2)	83 (13.2)	95 (11.9)		
Fit	12 (7.2)	133 (21.0)	145 (18.1)		
**Current Smoker**					
Yes	4 (2.4)	8 (1.3)	12 (1.5)	1.1	**0.221**
No	163 (97.6)	625 (98.7)	788 (98.5)		
Alcohol use^*^					
Current	3 (1.8)	1 (0.2)	4 (0.5)	7.8 ^a^	0.021^*^
Former	1 (0.6)	9 (1.4)	10 (1.25)		
Never	163 (97.6)	623 (98.4)	786 (98.25)		
III-Resilience^*^					
High	28 (16.8)	278 (43.9)	306 (38.25)	41	0.000^*^
Low	139 (83.2)	355 (56.1)	494 (61.75)		

*EPDS* Edinburgh Postpartum Depression Scale

^*^p ≤ 0.05

χ2 = Chi-square

^a^Fisher Test

Weak negative correlations were found between antenatal depression and the following independent variables: perceived social support (− 0.33, *p* < 0.01), emotional support of husband (− 0.31, *p* < 0.01), and resilience (− 0.22, *p* < 0.01). According to our data, no multicollinearity was detected; the computed Variance Inflation Factor (VIF) and tolerance were equal to 1. Many determinants were not found to be associated predictors of antenatal depression, such as low household income, gestational age, multigravida, and unplanned pregnancy. On the other hand, several other factors were identified as associated predictors for antenatal depression, as shown in Table 3.

**Table 3:** Predictors of Antenatal Depression through Logistic Regression Model (*n* = 800)

Explanatory variables	EPDS ≥ 13			
	OR [95% CI of Exp(B)]	***P*** value	AOR [95% CI of Exp(B)]	***P*** value
**Marital Conflict**				
Low	1		1	
High	6.2 [3.5–11]	0.000^*^	2.6 [1.1–3.1]	0.011^*^
**Financial distress**				
Low	1		1	
High	3.1 [1.7–7.6]	0.013^*^	2.6 [1.1–6]	0.011^*^
**Previous use medications related to mental illness**				
No	1		1	
Yes	36 [4.5–286]	0.001^*^	14 [1.2–172]	0.032^*^
**Fitness score**				
Active	1		1	
Fit	0.26 [0.14–0.4]		0.28 [0.17–0.8]	
Not Fit	7 [2–9]	0.000^*^	2.5 [1.5–5]	0.000^*^
**Stressful life event**				
No	1		1	
Yes	2.9 [1.5–5.7]	0.002^*^	2.9 [1.3–6.5]	0.008^*^
**Lack of emotional support**				
No	1		1	
Yes	11.4 [6.5–19]	0.000^*^	6.6 [3.2–13]	0.000^*^
**Fear of giving birth**				
Not relevant	1		1	
Highly relevant	4.7 [2.7–8.1]	0.004^*^	1.9 [1.1–3.5]	0.041^*^
**Concern about appearance**				
Not relevant	1		1	
Highly relevant	15 [5.9–37]	0.000^*^	3.2 [1.1–10]	0.011^*^
**Resilience**				
High	1		1	
Low	3.8 [2–5]	0.000^*^	2.1 [1.3–27]	0.002^*^
**Perceived social support**				
High	1		1	
Low	7.6 [4.6–11]	0.000^*^	3.2 [1.7–5.9]	0.000^*^
**Gestational Diabetes**				
No	1		1	
Yes	5 [1.8–13]	0.000^*^	2 [1.2–31.1]	0.002^*^

The model adjusted for Body Mass Index, unintended pregnancy, history of still birth, lack of postpartum support, comorbidities, trimesters, gravity, age, family size, intimate partner violence and low household income

*EPDS* Edinburgh Postpartum Depression Scale

*OR* Odd Ratio

*AOR* Adjusted Odd Ratio

^*^p ≤ 0.05

1 = Reference group

**Fig. 3:**
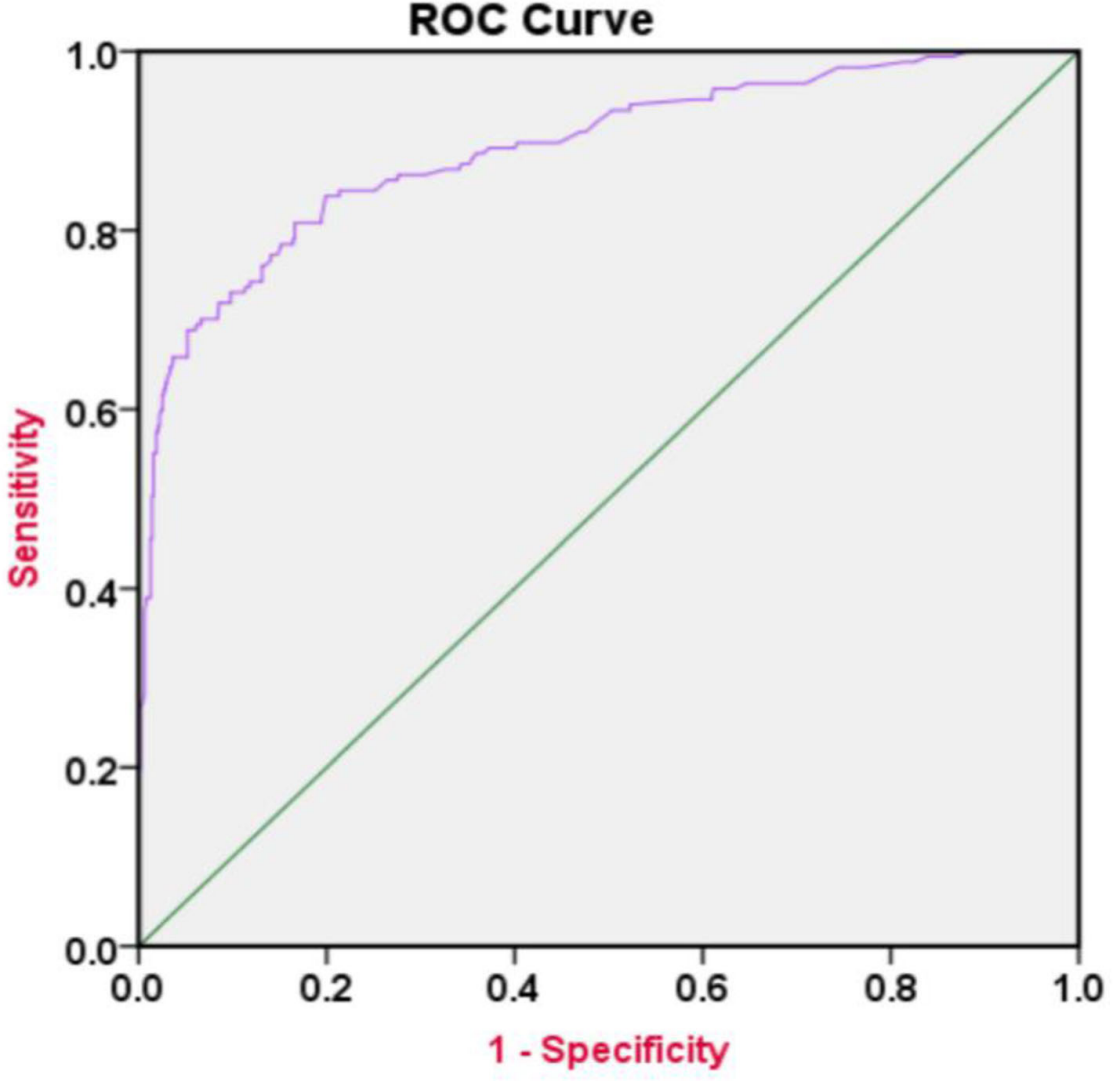
Predictive Probability demonstrated in the ROC-Curve

The Logistic Regression model discriminated depressed from non-depressed pregnant women with an accuracy of 89%. The computed Area Under the Curve was as high as (AUC = 0.89, CI [0.85 to 0.92]), sensitivity (53 to 94%), and specificity (96 to 99%) as seen in Fig. 3.

### Reliability of the tools

Based on our results, the utilized tools’ internal consistency appeared to be acceptable and indicate good reliability. This includes the Cronbach’s alpha of Ineffective Arguing Inventory tool (alpha =0.94), Resilience scale (RS-11) (alpha =0.91), Perceived Social Support tool (FSoz-6) (alpha = 0.89), ten items Pregnancy-Related Anxiety Revised Version Two (PRAQ-R2) (alpha = 0.87), EPDS tool (alpha = 0.85), and Intimate Partner Violence tool (HITS) (alpha = 0.76).

## Discussion

Our results showed that the prevalence of antenatal depression was around 20 % (*n* = 167, 20.9%) at 95% CI [18.2–23.8]. The percentage is remarkably lower than that reported in Jeddah, Saudi Arabia (*n* = 184, 57.5%) [22]. However, studies performed in Oman and Kuwait reported antenatal depression similar percentages to Qatar [35, 36]. On the other hand, our computed antenatal depression prevalence was higher than that reported in Australia (*n* = 1089, 6.2%) [37]. These comparisons are relevant as all the aforementioned studies were conducted at the primary health care level and utilized identical EPDS cut-off points.

‘Feeling overwhelmed’ was the most reported depressive symptom among pregnant women (*n* = 63, 7.9%), whereas ‘suicidal thoughts’ were only reported by a few participants (*n* = 3, 0.4%). These observations are contrary to the published findings in Saudi Arabia, where suicidal thoughts were the most reported symptoms in Jeddah among pregnant women [22]. Other Arab countries of the gulf focused on reporting the total score of depressive symptoms rather than pointing to a specific pathognomonic symptom [35, 36].

Multifactorial determinants showed a link to antenatal depression among pregnant women in Qatar. One of the significant determinants was the gestational age; pregnant women in their second trimester reported the highest depressive symptoms. A similar pattern was reported among pregnant women in Saudi Arabia, where the second trimester showed the highest depressive symptoms [38]. However, gestational age failed to predict antenatal depression in logistic regression, which indicates that this variable is not genuinely associated with antenatal depression. The finding may direct the policymakers to generalize depression screening to all the pregnant women with no specific gestational age, which is consistent with the USPTF that recommendation [37].

We found pregnancy-related anxiety an essential predictor for depressive symptoms among pregnant women in Qatar. These findings are similar to a previous study in Kuwait, where this variable was reported to be a vital predictor for antenatal depressive symptoms among pregnant women (AOR: 2.61; 95% CI [1.88 to 3.62]) [36]. Fear of giving birth construct successfully predicted antenatal depression by 2.6-folds (AOR: 2.63; 95% CI [2.39, 2.89]), similar to a previous study conducted in Finland [17]. Second, a low level of perceived social support proved to increase the probability of depression among pregnant women by seven-folds and succeeded in predicting our dependent variable (AOR: 3; 95% CI [1.7–5.9]). Our outcomes were in line with studies conducted among pregnant women from Jamaica and England [19, 22].

Upon exploring the biological risk, previous use of mental health medications found to predict antenatal depression directly. Our findings opposed a previous study published in 2004 that showed biological factors as insignificant predictors for antenatal depression [12]. Women who discontinued anti-depressant therapy during pregnancy were more likely to deteriorate. Additionally, gestational diabetes showed to predict antenatal depression, which was consistent with the results revealed by a structural equation model conducted in a prospective cohort study in 2014 where gestational diabetes was a significant predictor for depressive symptoms among pregnant women [39].

Biopsychosocial determinants were at the forefront of the predictors, which once combined could predict as high as 92% of the outcome. Indicating the crucial role of adopting a holistic approach in assessing all significant predictors of antenatal depression in primary health care settings.

### Strengths and limitations

To our knowledge, this study is the first population-based research to explore the prevalence and determinants of antenatal depression in Qatar. One of this study’s strengths is testing sampling bias through bootstrapping that showed negligible bias and allowed results to be generalized for Qatar’s whole population. Furthermore, our large representative sample size ensures acceptable external validity. Additionally, we utilized a triple source of information (medical records, interviewer-administrated questionnaire, and self-report) for data collection. Those combined methods strengthen the findings and rule out information bias from the study.

Our study provided valuable insights into the determinants of depressive symptoms among pregnant women in Qatar, but it was not without limitations. For instance, for measuring the prevalence of depressive symptoms in pregnancy, a self-reported screening tool (EPDS) was utilized instead of a structured diagnostic interview operated by a psychiatry specialist due to a lack of resources. Four possible outcomes could be retrieved from our finding [true and false positives, true and false negatives]. Another limitation is that the causal effect could not be confirmed as the determinants were measured simultaneously with the outcome. Therefore, temporality was absent which indicates that the causation effect must be interpreted with caution.

## Conclusions

Our results demonstrated that two in ten women might experience depression during pregnancy. Clinicians must adopt a holistic approach in identifying depressed pregnant women, focusing on significant biopsychosocial predictors, specifically a previous history of mental illness, current gestational diabetes, low resilience, low perceived social support, and high pregnancy-related anxiety.

## Data Availability

The datasets used and/or analysed during the current study are available from the corresponding author on reasonable request.

## Abbreviations

WHO: World Health Organization
USPTF: United State Preventive Task Force
PHCC: Primary Health Care Corporation
EPDS: Edinburgh Postpartum Depression Scale
PRAQ-R2: Pregnancy-Related Anxiety Revised Version Two
HITS: Hurt Insult Threaten and Scream
RS-11: Resilience Scale
BMI: Body Mass Index
QR: Qatari Ryal
OR: Odd Ratio
AOR: Adjusted Odd Ratio
CI: Confidence Interval
SD: Standard deviation
VIF: Variance Inflation Factor
AUC: Area Under the Curve
